# Optimized Production of Xylitol from Xylose Using a Hyper-Acidophilic *Candida tropicalis*

**DOI:** 10.3390/biom5031979

**Published:** 2015-08-19

**Authors:** Elena Tamburini, Stefania Costa, Maria Gabriella Marchetti, Paola Pedrini

**Affiliations:** Department of Life Sciences and Biotechnology, University of Ferrara, Via L. Borsari 46, Ferrara 44121, Italy; E-Mails: stefania.costa@unife.it (S.C.); mhm@unife.it (M.G.M.); pdp@unife.it (P.P.)

**Keywords:** xylitol, xylose, fed batch fermentation, *Candida tropicalis*, hyper-acidophylic

## Abstract

The yeast *Candida tropicalis* DSM 7524 produces xylitol, a natural, low-calorie sweetener, by fermentation of xylose. In order to increase xylitol production rate during the submerged fermentation process, some parameters-substrate (xylose) concentration, pH, aeration rate, temperature and fermentation strategy-have been optimized. The maximum xylitol yield reached at 60–80 g/L initial xylose concentration, pH 5.5 at 37 °C was 83.66% (w/w) on consumed xylose in microaerophilic conditions (k_L_a = 2·h^−1^). Scaling up on 3 L fermenter, with a fed-batch strategy, the best xylitol yield was 86.84% (w/w), against a 90% of theoretical yield. The hyper-acidophilic behaviour of *C. tropicalis* makes this strain particularly promising for industrial application, due to the possibility to work in non-sterile conditions.

## 1. Introduction

Xylitol, a five-carbon sugar alcohol, occurs widely in nature but it is also a normal intermediate in humans [1]. Because of these characteristics and benefits, it is widely and increasingly used in the food and confectionary industries [2], and its dietary and chemical properties have been extensively studied [3].

Primary interest in xylitol centers on its properties and potential uses as an alternative sweetener. Contrary to other widespresd non-caloric sweeteners, such as saccharine or aspartame, d-xylitol has many properties similar to those of sucrose. It dissolves readily in water, it is as sweet as sucrose, despite having only approximately two-third of its calories, and is approximately twice as sweet as sorbitol and nearly three times as sweet as mannitol [4].

Xylitol also has greater negative heat of solution, providing a distinct cooling sensation (also known as “cold”, “fresh” or “minty”) and more anticariogenic properties than other common polyols [5]. Since a research study conducted in Finland evaluating the effectiveness of xylitol on dental plaque reduction in 1970, xylitol has been widely researched and globally accepted as a natural sweetener approved by the U.S. Food and Drug Administration (FDA) and the American Academy of Pediatric Dentistry [6]. By now, more than 35 countries have approved the use of xylitol in foods, pharmaceuticals, and oral health products, principally in chewing gums, toothpastes, syrups, and confectioneries. Regular xylitol consumption has been defined as 5–7 g of daily consumption of xylitol at least three times a day for human adults [7]. Furthermore, it may be used clinically as a sugar substitute for diabetics or of glucose-& phosphate-dehydrogenase deficient population, due to its slow adsorption and entry into metabolic pathways independently of insulin and without causing rapid fluctuation of blood glucose [8].

Xylitol is a biomolecule widely present in plants, especially in certain fruits and vegetables (*i.e*., raspberry, strawberry, yellow plum, endive). However, the extraction process from such sources is uneconomical, due to the relatively low xylitol content, generally below 1%, on dry weight basis [9]. Therefore, on industrial scale, xylitol is mostly produced through a chemical method, which is based on catalytic xylose dehydrogenation [10]. The method is expensive, energy intensive, and environmentally hazardous because of the use of a toxic Raney nickel catalyst and high-pressure hydrogen gas [11]. These drawbacks motivated researchers to seek alternative procedures. Biotechnological approaches for xylitol production, using xylose-fermenting yeasts, and employing NADPH-dependent xylose reductase to reduce xylose to xylitol, are safe, environmentally friendly, and highly productive [12]. Microbial xylitol production has been considered more favorable and sustainable for industrial applications because, as it is well-known, the process can be conducted under mild conditions such as atmospheric pressure and ambient temperature [13]. Among microbes for xylitol production, yeast with the use of xylose as elective substrates, look like the best candidates. In particular, several yeast strains described within the genus Candida, belonging to the species *C. boidinii*, *C. guillermondii*, *C. parapsilosis*, and *C. tropicalis*, have been extensively studied in this regard [14].

Although *Candida* spp. could be considered as opportunistic pathogens, it is currently used in industry because of its general high xylitol yield, due to their high xylose uptake rate and xylitol production capacity [15]. Great efforts have been made to improve or optimize biological production of xylitol by yeasts because of high demand from the pharmaceutical and food industries [16,17,18].

Xylitol accumulation in *Candida* spp. is influenced by a number of process conditions, such as temperature, pH, and oxygen transfer rate [19]. Despite the large information available over the years on the effect of these parameters on xylitol production by Candida yeasts [20,21,22], only little research has been done on *C. tropicalis* DSM 7524 [23,24]. Because of its being particularly acidophilic, *C. tropicalis* is particularly promising for industrial application, due to the possibility of working and adapts to be used in non-sterile conditions.

During the research presented in this article, several preliminary batch fermentation, both in flask and on lab fermenter scale were carried out in order to clarify the effects of pH, temperature, aeration rate and substrate concentration on xylitol production from xylose. Because of a high concentration of a polyhydroxyl compound, as xylitol, increases the osmotic pressure, which, in turn, affects metabolism and interferes with the membrane transport system of the cells, the use of a fed-batch strategy will be also described.

## 2. Results and Discussion

### 2.1. Effect of Temperature

**Figure 1:**
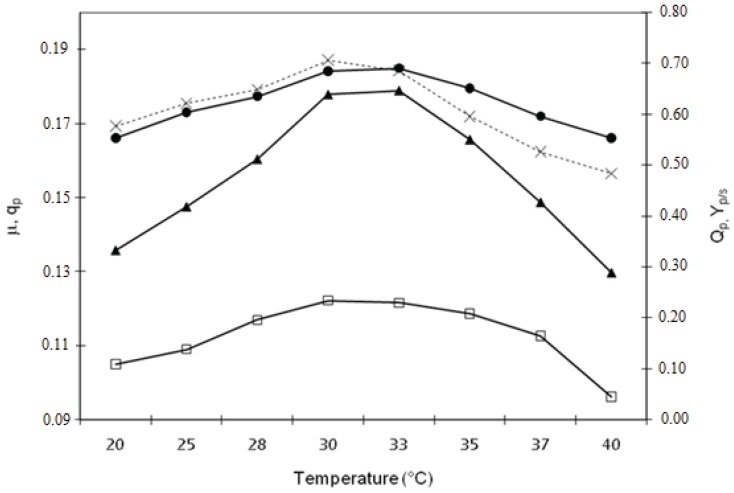
Effect of temperature on fermentation. Specific growth rate (●), µ; specific production rate of xylitol (□), q_p_; volumetric production rate (▲), Qp; and xylitol yield on xylose consumed (×), Y_p/s_.

**Figure 2:**
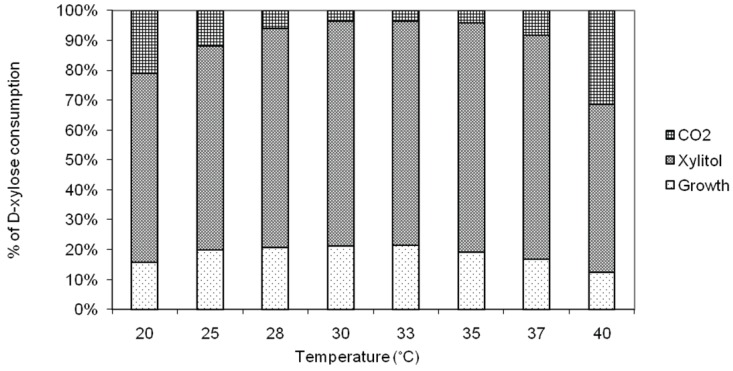
Percentage of xylose consumption in the metabolism of *C. tropicalis* DSM 7524 at different temperatures.

Growth rate and xylitol production are temperature-dependent because both the ability of the cell to sequester xylose from the environment (regulated by its affinity with the transport proteins in the membrane) and the efficiency of intracellular enzymes (regulating the assimilation of xylose) are temperature-dependent [25]. This implies that, decreasing temperature, any substrate taken up by some form of active transport, as xylose, becomes increasingly less available in Candida yeasts. On the other hand, as temperature rises, limits on increases are imposed by the thermal inactivation of key cellular components. The effects of temperature from 20 °C to 40 °C on the specific growth rate (µ), specific productivity (q_p_), volumetric productivity (Q_p_) and xylitol yield on consumed substrate coefficient (Y_p/s_) were investigated. As shown in Figure 1, the fermentation was almost temperature-independent when the yeast was cultured in a range between 29 °C and 34 °C, but above 35 °C and below 28 °C the xylitol yield, Q_p_ and Y_p/s consumed_ sharply decreased. Figure 2 shows percentage of xylose converted in xylitol and CO_2_ after 56 h fermentation at different temperatures reported. Since no ethanol production took place, data show that, between 28 °C and 37 °C, more than 90% (w/w) of xylose was consumed for xylitol production. Moreover, the best combination of xylose consumption, which could maximize xylitol yield and minimize growth and respiration, was obtained narrowing the range between 29 °C and 34 °C. In absence of respirometric measurements, the amount of CO_2_ was approximately estimated assuming that the remaining amount of xylose not consumed for cells growth and for xylitol production was used by the yeast for respiration.

### 2.2. Effect of External pH

**Figure 3:**
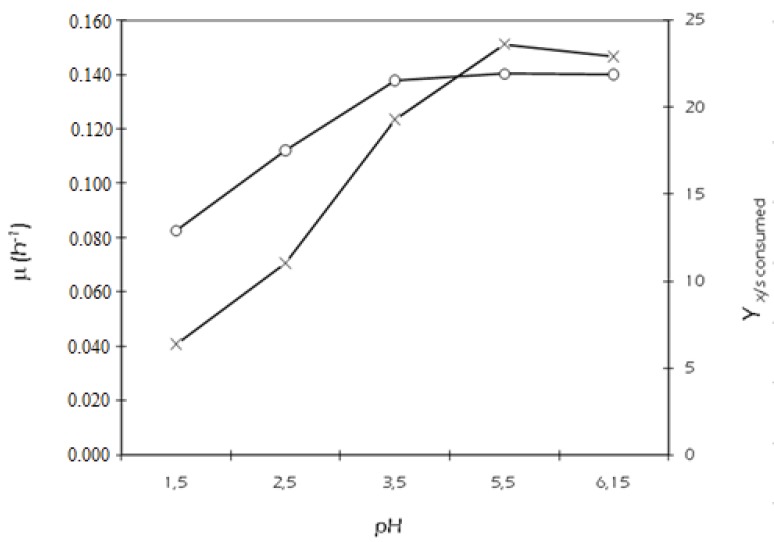
Influence of variable pH on fermentation. Specific growth rate (○), µ; and biomass yield on xylose consumed (×), Yx/s consumed.

**Figure 4:**
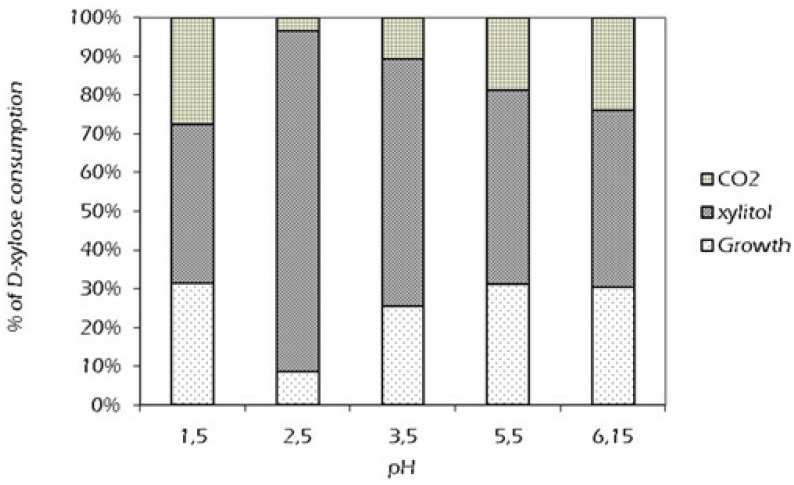
Percentage of xylose consumption in the metabolism of *C. tropicalis* DSM 7524 at different pH levels.

The experiments were run in flasks at various pH levels at a temperature of 32 °C (a mean value chosen in the interval 29–34 °C) to examine the effect of pH on specific growth rate and product yields. In each experiment, the initial xylose concentration was 30 g/L. The maximum specific growth rate and the yield coefficient of biomass on initial xylose occurred at pH 5.5 (Figure 3), but the kinetic results revealed that the best productive performances in xylitol were undoubtedly obtained at pH 2.5 (Table 1). As before, carbon balances were used to illustrate the effect of pH on product yields. Figure 4 summarizes the percentage of xylose recovered in each product at each pH. At pH 2.5, 88.05% of the total xylose assimilated was converted to xylitol by the relatively small amount of cells which are able to survive at such a low pH level. The mechanism of xylitol formation in *C. tropicalis* is a bioreduction, where the first metabolic step is the xylose uptake through the plasma membrane. The electrochemical H^+^ gradient across the membrane required for the uptake of sugars could be affected by external pH variations, making xylose transport from the bulk to inside the cell the rate-limiting step for fermentation. Our results seem to indicate that *C. tropicalis* DSM 7524 does not share a common characteristic with other Candida species or other xylose-grown yeasts, as *C. shehatae* [26] and *D. hansenii* [27], having the best xylitol yield at pH 5.5. Such a hyper-acidophilic behavior could be explained by either a positive pH incidence on the redox balance of the bioreduction of xylose to xylitol, or an effective cell internal homeostasis in counterbalancing the low external pH, which turns a significant external decrease into a slight internal consequence.

**Table 1:** Effect of pH on the main kinetic parameters.

pH	*Y_p/s initial_*	*Y_p/x_*	*q_s_* (g/g h)	*Q_p_* (g/L h)	*q_p_* (g/g h)
1.5	0.08	1.57	0.162	0.096	0.060
2.5	0.61	6.40	0.349	0.815	0.246
3.5	0.32	3.30	0.199	0.515	0.116
5.5	0.25	2.70	0.163	0.392	0.074
6.15	0.16	1.80	0.140	0.385	0.070

### 2.3. Effect of Initial Xylose Concentration on Xylitol Yield

The effect of the xylose concentration on xylitol productivity indexes was investigated in order to determine the optimum substrate concentration for fed-batch fermentations. The effect of the initial substrate concentration is particularly relevant in the design of a fermentation strategy, because this parameter directly influences xylose metabolism and xylitol extracellular secretion.

Batch runs were performed, modifying the initial xylose concentration (15–300 g/L) and, evaluating the xylose consumed, the specific and volumetric production of xylitol and the xylitol yield from xylose (Table 2). Under DO (dissolved oxygen) conditions maintained (3.5% air saturation), at high concentrations (more than 200 g/L) xylose inhibited the growth of xylitol-accumulating yeasts demonstrating substrate inhibition effect, while at low concentration (less than 50 g/L) the production of xylitol dropped, and a considerable fraction of xylose is respired. Consequently, maximum xylitol productivity was obtained at intermediate starting xylose levels (60–200 g/L), depending on the microorganism [28,29]. The results demonstrated that our Candida had a maximum of xylitol yield, both on initial and consumed xylose (Y_p/s initial_ = 71.52%–54.99% and Y_p/s consumed_ = 71.52%–83.66%, respectively), in the range between 60 g/L and 80 g/L of substrate starting level. Moreover, the specific xylose consumption rate (q_s_ = 0.063–0.083 g/g h), and both the specific (q_p_ = 0.051–0.059 g/g h) and volumetric (Q_p_ = 0.512–0.630 g/L h) xylitol production rate reached a maximum in correspondence of the same xylose concentration interval. Xylose bioreduction to xylitol showed a progressive decrease in productivity besides 80 g/L, which led to a dramatic drop on the xylitol yields. These results seem to contradict previous findings reported in Tamburini *et al.* [30], where maximum yields of xylitol obtained on initial xylose concentration of 50–70 g/L were 38%–42% respectively. However, it is worth noting that our case experiments were carried out in fermenter under controlled values of air saturation, temperature and pH all along the fermentations. This allowed for the maintenance of cultures at their best conditions, impossible to be guaranteed in an Erlenmeyer flask. Notably, small-scale experiments permitted exclusion from this study were the presence of co-substrates of fermentation (mono- and di-saccharides) as possible further positive variables to be investigated for increasing xylitol yields.

**Table 2:** Effect of initial xylose concentration (S) on the main kinetic parameters.

*S* (g/L)	µ (1/h)	*Y_p/s initial_*	*Y_p/s consumed_*	*q_s_* (g/g h)	*Q_p_* (g/L h)	*q_p_* (g/g h)
15	0.031	30.13	30.13	0.027	0.073	0.008
30	0.034	33.80	33.80	0.035	0.141	0.012
40	0.034	43.78	48.62	0.035	0.204	0.017
50	0.034	49.18	52.67	0.048	0.297	0.025
60	0.030	71.52	71.52	0.083	0.512	0.059
70	0.033	65.76	74.35	0.068	0.557	0.051
80	0.033	54.99	83.66	0.067	0.616	0.056
100	0.017	42.33	67.82	0.029	0.236	0.020
150	0.010	25.17	64.10	0.017	0.142	0.011
200	0.007	12.50	35.71	0.015	0.070	0.005
300	0.004	3.33	29.41	0.020	0.028	0.006

**Figure 5:**
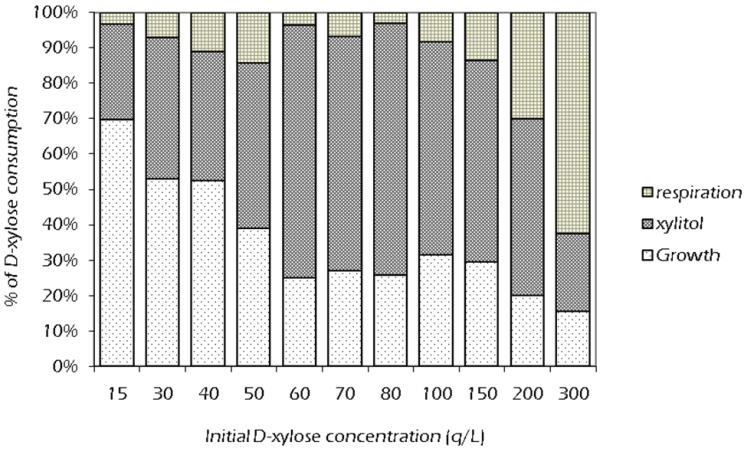
Percentage of xylose consumption in the metabolism of *C. tropicalis* DSM 7524 at different temperature.

Following the results, carbon material balances for the consumption of xylose were performed (Figure 5). Despite what reported in previous literature [23,31], as the initial xylose concentration increased, the xylose consumed for biomass growth progressively decreased, whereas the fraction for respiration raised, and maintained almost constant the percentage used for xylitol production. This means that, at higher starting carbon source levels, the fermentation was progressively inhibited and a larger amount of xylose was consumed to get enough energy for respiration and ATP production, in order to counterbalance the higher osmotic pressure derived from the higher substrate concentration.

### 2.4. Set Up of Fed-Batch Fermentation

The fed-batch strategies was set up to optimize the final xylitol concentration. In fact, xylitol, like other metabolites such as alcohol or organic acids, is secreted by diffusion or passive transport using the concentration gradient across the membrane. Therefore, an extracellular xylitol concentration as low as possible must be maintained. The fermentation conditions were established on the basis of the results previously obtained, *i.e*., 32 °C, xylose initial concentration of 80 g/L and pH 5.5 during the cell growth (batch phase). The pH was lowered to 2.5 during the xylitol bioreduction (fed phase). During the batch phase the dissolved oxygen (DO) was maintained at saturation level with cascade mode, assisting the aerobic metabolism, whereas during the fed phase, the oxygen supply rate was controlled by a K_L_a value of 2·h^−1^, obtained with no air flow and 150 rpm stirring (DO <1% of saturation). The feeding of xylose solution was regulated with a peristaltic pump based on µ and q_s_ values and in order to maintain the concentration of substrate around 80 g/L and the biomass concentration at a constant value. The fermentations were run batchwise up to 16 h to achieve a high cell mass concentration. The fed mode of operation was started by feeding the xylose solution when the starting concentration was completely depleted. In 81 h of fed-batch run (16 h batch and 65 h fed) the total xylitol produced was 71.34 g/L, on a xylose concentration of 82.11 ± 3.78 g/L, reaching a yield of 86.84% (w/w), with a volumetric productivity of xylitol of 0.79 g/L h. 

## 3. Experimental Section

### 3.1. Strain and Chemicals

All chemicals were purchased from Sigma-Aldrich (Steinheim, Germany) unless otherwise stated. The strain *Candida tropicalis* DSM 7425 was purchased from the German Resource Centre for Biological Material (DSMZ, Braunschweig, Germany). The strain was routinely cultured in aerobiosis at 35 °C in Yeast Mold medium (YM, BD Difco, Franklin Lakes, NJ, USA), containing: 20 g/L glucose, 5 g/L bactopeptone, 3 g/L yeast extract, 3 g/L malt extract, The yeast was cultured in a 500 mL Erlenmeyer flasks containing 100 mL of YM medium at 35 °C and pH 5.5. After 20 h incubation at 200 rpm, the fresh cells were harvested by sterile centrifugation and used as inoculum.

Xylitol production was performed in a xylose-containing synthetic medium (hereinafter referred to as XSM), with the following composition: 12.5 g/L (NH_4_)_2_SO_4_, 3 g/L KH_2_PO_4_, 1.25 g/L MgSO_4_, 0.45 g/L ZnSO_4_·7H_2_O, 0.45 g/L CaCl_2_·2H_2_O, 0.3 g/L CoCl_2_·6H2O, 0.3 g/L CuSO_4_·5H2O, 0.3 g/L FeSO_4_·7H_2_O, 0.1 g/L KI, and 0.015 g/L EDTA. The preliminary experiments at variable pH, variable temperature and variable starting xylose concentration (15 to 300 g/L) were carried out in 500 mL Erlenmeyer flasks containing the above culture medium (150 mL) at 200 rpm for 1–4 days using an amount of inoculum to reach an initial optical density of 0.25 ± 0.05 AU (λ = 600 nm). The fermentation at variable temperatures were performed at a starting pH of 5.5 while the experiments at variable pH at 32 °C.

### 3.2. Xylitol Production

In both flasks and bioreactor experiments, XSM medium was inoculated with YM cultures, grown for 16 h at 30 °C and 200 rpm in shaken flasks. An appropriate volume of inoculum was utilized, in order to obtain and initial turbidity (λ = 600 nm) of 0.25 ± 0.50 AU. Flask cultures in XSM medium were carried out at different temperatures, initial pH values, and d-xylose concentration. Unless otherwise stated, temperature was 32 °C, initial pH was 5.5, and xylose was 80g/L.

Bioreactor batch cultures were performed in a benchtop apparatus (Solaris, Porto Mantovano, Italy) containing 2.5 L of XSM. The culture was aerated with 0.5 v/v/min air; stirring was regulated in the range from 150 to 900 rpm to prevent the DOT from decreasing below 3.5%. Once the best temperature was determined (29–34 °C), external pH (5.5 to 2.5) and xylose initial concentration (80 g/L), and fed-batch fermentations strategy were assessed. Fed-batch experiments were initiated batchwise in 2 L of XSM. When biomass reached exponential phase and the residual concentration of xylose decreased to 40 g/L, which was predicted from previous batch fermentations and confirmed by off-line assay, a fed-batch process with a feeding of highly concentrated xylose solution into a reactor was conducted. The solution of xylose was fed continuously using a peristaltic pump (Shimadzu) by adjusting the pump speed in the range of 15–60 mL/h, as to increase and maintain the xylose concentration at 80 g/L. The aeration rate and stirring rate were set up in cascade mode so automatically adjusting the dissolved oxygen (DO) concentration at 3.5% air saturation.

### 3.3. Analytical Methods

Xylose, xylitol and ethanol were quantified using a HPLC device (Shimadzu, Kyoto, Japan), equipped with refractive index detector and ion-exclusion column Aminex HPX-87H (Bio-Rad Laboratories, Redmond, WA, USA). Isocratic elution was carried out at 40 °C with 8 mL/min of 50 mM H_3_PO_4_.

Growth was monitored by measuring the turbidity at 600 nm. Biomass concentration (dry weight, DW) was determined gravimetrically after drying it overnight at 105 °C on a pre-weighed 0.2 μm filter.

### 3.4. Calculation of Yields, Kinetic Parameters and Carbon Material Balances

All yields (Y_p/s consumed_, Y_p/s initial_, Y_p/x_) were calculated on a mass basis rather than a concentration basis in order to minimize errors that may results from volume changes due to evaporation, sample withdrawal, addition of base for pH control, and addition of culture medium during fed-batch culture. Specific rates of xylose consumption (q_s_) were calculated as the xylose consumption divided by the average cell mass and the time of fermentation. Specific (q_p_) and volumetric (Q_p_) xylitol production were calculated as the amount of xylitol produced divided by the average cell mass, or volume of the culture media respectively, and time.

The carbon material balances have been performed based on the models reported by Roels [32] and Barbosa [33] for the fermentation of xylose by yeast. Based on this mode of operation, the theoretical yield is 0.9 mol of xylitol per mol of xylose utilized. The relevant equations are reported in the above-mentioned papers.

## 4. Conclusions

Xylitol production by *C. tropicalis* DSM 7524 has been investigated under microaerophilic conditions varying alternatively temperature, pH and concentration of the carbon source. Combining the above mentioned parameters (temperature 32 °C, 80 g/L of starting xylose concentration, pH 2.5), a maximum of xylitol productive performances was reached. The results obtained have been utilized to set up a fed-batch fermentation strategy over 81 h, where the final best xylitol yield was of 86.84% (w/w), against a 90% (w/w) of theoretical yield. This study has demonstrated that *C. tropicalis* DSM 7524 could be efficiently employed in industrial processes for producing xylitol, and it could represent an impressive step forward towards the sustainable and efficient use of resources.
